# Highly stable *Saccharomyces cerevisiae* L-BC capsids with versatile packing potential

**DOI:** 10.3389/fbioe.2024.1456453

**Published:** 2024-09-25

**Authors:** Enrika Celitan, Ramunė Stanevičienė, Elena Servienė, Saulius Serva

**Affiliations:** ^1^ Laboratory of Nucleic Acid Biochemistry, Department of Biochemistry and Molecular Biology, Life Sciences Center, Vilnius University, Vilnius, Lithuania; ^2^ Laboratory of Genetics, Nature Research Centre, Vilnius, Lithuania

**Keywords:** virus-like particles, encapsulation, particle stability, L-BC virus, *Saccharomyces cerevisiae*

## Abstract

Virus-like particles (VLPs) are promising nanoscaffolds in development of vaccines and nanodelivery systems. Along with efficient production in various expression systems, they also offer extensive functionalization options. Nevertheless, the ultimate integrity of VLPs is an important burden for the applicability in nanobiotechnology. In this study, we characterize the *Saccharomyces cerevisiae* L-BC VLPs synthesized and purified from *Escherichia coli* and *Saccharomyces cerevisiae* cells. The particles exhibited prominent size stability in buffers within a range of ionic strength conditions, pH environment and presence of magnesium ions during the long-term storage at temperatures up to 37°C. Bacteria-derived particles exhibited alleviated stability in acidic pH values, higher ionic strength and temperature compared to yeast-derived particles. Taking advantage of gene engineering, 120 copies of red fluorescent protein mCherry were successfully encapsulated into both preparations of L-BC VLPs, while passive diffusion enabled encapsulation of antimicrobial peptide nisin into the yeast-derived unmodified VLPs. Our findings indicate that L-BC VLPs generally exhibit high long-term stability under various conditions, while yeast-derived L-BC VLPs are more stable under the elevated temperatures than bacteria-derived particles. Stability studies and encapsulation of particles by different molecules involving alternative strategies delineate the L-BC VLP potential to be developed into versatile nanodelivery system.

## 1 Introduction

Since the discovery of the molecular composition of viruses, their structure is in the focus of close investigation (Rossmann, 2013). In the late 50s, the use of electron microscopy for the investigation of HeLa cells infected with poliovirus resulted in the discovery of: a) complete particles, b) partially assembled shells of subunits - incomplete particles and c) empty shells or “ghost” particles (Horne and Nagington, 1959). At that time the biological nature of empty poliovirus particles was unknown, however corresponding fractions were previously separated in a sucrose density gradient. It was assumed, that complete particles may be infective virus, while incomplete or empty particles – non-infectious virions (Horne and Nagington, 1959; Le Bouvier et al., 1957). Later it was shown that structural proteins of many viruses can spontaneously form both virions and virus-like particles (VLPs) (Grgacic and Anderson, 2006).

VLPs are nanosized structures composed of self-assembled viral proteins closely resembling the structure of native virus, considered non-infectious due to the lack of viral genetic material (Nooraei et al., 2021; Zhang et al., 2023; Kim et al., 2023; Deshayes et al., 2022). These protein cages can be readily synthesized in various expression systems, such as bacteria, yeasts, plant, insect, mammalian cells, or even cell-free systems (Wahome et al., 2006; Kim and Kim, 2017; Scotti and Rybicki, 2013; Fernandes et al., 2013; Wu et al., 2010; Spice et al., 2020; Sheng et al., 2017). All expression systems have specific advantages and limitations, such as the production cost and time, protein yield, appropriate protein folding, and availability of post-translational modifications. Therefore, the selection of proper expression system is crucial for production of VLPs with properties important for future applications (Fuenmayor et al., 2017; Zeltins, 2013).

Virus-like particles are non-infectious, readily synthesized, biodegradable, and naturally biocompatible; generally, they are structurally flexible (Chung et al., 2020; Tariq et al., 2022). Incorporation of various molecules, both of biological and synthetic origin, into the VLPs can be achieved by several strategies: passive-diffusion of small molecules through capsid pores (infusion), genetic engineering, chemical conjugation, or controlled assembly of capsid proteins (Chung et al., 2020; McNeale et al., 2023). These modifications can be applied before and/or after the synthesis of VLPs to obtain more stable particles with better functional properties (Mejía-Méndez et al., 2022). The desired properties of VLPs mainly depend on the field of application. Developing of VLP-based vaccines requires the property of the particles to elicit a strong cellular and humoral immune response, while in developing VLP-based nanodelivery system it is essential to achieve targeted delivery; therefore, immunogenicity generally should be avoided (Dai et al., 2018; He et al., 2022). It is also necessary to ensure the stability of particles since aggregation of VLPs is a common issue that affects immunogenicity, antigenicity, and dose formulation; particle size stability is of significant importance for an efficient delivery system (Chen et al., 2015; Srivastava et al., 2023; Masarudin et al., 2015).

For the development of nanodelivery systems, various virus-like particles are explored in the search for the most desirable property set. A non-infectious yeast dsRNA virus L-BC was chosen for the development of VLPs. L-BC virus is one of two totiviruses found in most *S. cerevisiae* strains, lacking negative effect on the host cell (Ribas and Wickner, 1996; Schmitt and Breinig, 2002). L-BC virus bears a double-stranded RNA genome (4.6 kb), which encodes the major capsid protein Gag (78.3 kDa) and RNA-dependent RNA polymerase Pol, synthesized only as Gag-Pol fusion protein (171.5 kDa) (Park et al., 1996). The dsRNA genome of L-BC virus is packed in capsid with icosahedral symmetry (38.5 nm) composed of 120 Gag proteins, a few of which fused with Pol (Grybchuk et al., 2022).

In this study, we demonstrate that bacteria *Escherichia coli* and yeast *Saccharomyces cerevisiae* are suitable expression systems for the synthesis of L-BC Gag protein and subsequent self-assembly into VLPs. To evaluate the size stability of purified L-BC VLPs in different storage buffers and temperature conditions, dynamic light scattering (DLS), transmission electron microscopy (TEM), and fluorescent thermal shift assay (FTSA) methods were employed. We demonstrate that both bacteria- and yeast-derived L-BC VLPs are highly stable in various conditions for prolonged periods, while yeast-derived particles observe better stability properties under elevated storage temperatures. The fusion with capsid protein led to the successful encapsulation of 120 copies of mCherry protein inside the VLP, making it exceptionally bright yet stable. Passive diffusion technique enabled loading of antimicrobial peptide nisin Z inside the VLP, conferring the antibacterial activity, further showcasing the denoted potential of L-BC VLPs as a versatile nano-packaging and delivery system.

## 2 Materials and methods

### 2.1 Strains

Bacterial and yeast strains used in this study are summarized in Table 1.

**TABLE 1:** Strains used in this study.

Strain	Species	Genotype	Source
DH10B	*Escherichia coli*	F– endA1 deoR + recA1 galE15 galK16 nupGrpsL Δ(lac)X74 φ80lacZΔM15 araD139 Δ(ara,leu)7,697 mcrA Δ(mrr-hsdRMS-mcrBC) StrR λ–	Thermo Fisher Scientific
BL21-AI		F- ompThsdSB (rB-mB-) gal dcmaraB::T7RNAPtetA	Invitrogen
BY4741	*Saccharomyces cerevisiae*	MATa, his3∆1, leu2∆0, met15∆0, ura3∆0, ScV-LA1, ScV-LBC1	Thermo Fisher Scientific
BY4741 [LA-LBC-]		MATa, his3∆1, leu2∆0, met15∆0, ura3∆0, cured of ScV-LA1 and ScV-LBC1 viruses	This laboratory

### 2.2 Construction of an expression vectors

All enzymes, molecular mass standards, and kits for DNA manipulations were purchased from Thermo Fisher Scientific Baltics (Vilnius, Lithuania) and used following the manufacturer’s recommendations. Oligonucleotides were obtained from Metabion (Planegg, Germany).

Genomic dsRNA of the native L-BC virus was extracted from *S. cerevisiae* strain BY4741 as described previously (Lukša et al., 2017). Reverse transcription of L-BC virus GAG gene was performed by RevertAID reverse transcriptase and L-BC-dir-3 (5′-CCG​TCT​CAC​**ATG**​TCC​TCT​CTG​TTA​AAT​TCA​TTA​C-3′), L-BC-rev-2 (5′-**CTA**​TTC​TAT​ATC​CGG​TGG​AAG-3′) oligonucleotide primers using extracted dsRNA as a template. ATG and termination codons are indicated in bold. The cDNA amplification of GAG gene was performed by Phusion High-Fidelity DNA Polymerase using L-BC-dir-3 and L-BC-rev-2 primers.

For Gag protein expression in bacteria, PCR product was digested with Esp3I restriction endonuclease and inserted by treating with T4 DNA ligase into the NcoI- and Ecl136II-digested vector pET28a (Shilling et al., 2020) under the control of T7 promoter. Resulted expression vector pET28a-LBC-gag was propagated in E. *coli* DH10B cells and the sequence was verified by DNA sequencing. For Gag protein expression in yeast, the same Esp3I-digested PCR product was blunted by Klenow fragment and inserted by T4 DNA ligase treatment into the BglII-digested and blunted vector pFX7 (Sasnauskas et al., 1999) under the control of hybrid GAL10-PYK1 promoter. The sequence of expression vector pFX7-LBC-gag was verified by DNA sequencing.

For Gag and mCherry fusion protein expression in bacteria, we used pYAK3-LBC-gag+mCherry plasmid constructed previously in our laboratory (unpublished). It was digested with EcoRI and XhoI restriction endonucleases and treated by T4 DNA ligase, so that gene coding for mCherry was inserted into the pET28a-LBC-gag vector digested with the same endonucleases. The sequence of pET28a-LBC-gag + mCherry was verified by DNA sequencing. For Gag-mCherry fusion protein expression in yeast, pYAK3-LBC-gag+mCherry plasmid was digested with BcuI and BshTI restriction endonucleases. Gag-mCherry encoding fragment was blunted using the Klenow fragment and inserted into the BglII-digested and blunted vector pFX7. The sequence of pFX7-LBC-gag + mCherry vector was verified by DNA sequencing.

### 2.3 Purification of recombinant Gag and Gag-mCherry proteins from bacteria

Synthesis of L-BC Gag and Gag-mCherry fusion protein was performed in *E. coli* BL21-AI cells using expression vector pET28a-LBC-gag and pET28a-LBC-gag + mCherry, respectively. Selection of transformants resistant to kanamycin was carried out on the Luria-Bertani (LB) (1% tryptone, 1% NaCl, 0.5% yeast extract) agar plates (2% agar) supplemented with 50 μg/mL kanamycin. Transformed colonies were grown in 600 mL LB medium supplemented with 50 μg/mL kanamycin to an OD600 of 0.6–0.8 before the induction with 0.1% (w/v) L-arabinose and 0.1 mM IPTG for 4 h at room temperature. After induction, bacterial cells were harvested by centrifugation at 5,000×g for 10 min at 4°C and frozen at −20°C if purification was carried out later. Harvested cells were resuspended in 10 volumes (w/v) of lysis buffer (100 mM Tris-HCl pH 8.0, 150 mM NaCl, 1 mM EDTA) with 1 mM phenylmethanesulfonylfluoride (PMSF) and 0.1 mg/mL lysozyme. The cell suspension was sonicated on ice at 40% amplitude 5–7 times for 30 s, with 30 s breaks between the sonications using a Vibra-cell VCX-130 ultrasonic processor (Sonics). Cell debris and insoluble protein fraction were separated from the soluble protein fraction by centrifugation at 15,000×g for 15 min at 4°C. The soluble protein fraction was diluted by lysis buffer to 15 mL and transferred to 45% (w/v) sucrose solution in lysis buffer. Sedimentation of VLPs was carried out by ultracentrifugation at 76,000×g for 16–18 h at 4°C using MSE MS60 ultracentrifuge (Sanyo) with TST 28.38 rotor (Kontron). The resulting pellet was resuspended in 300 µL lysis buffer and sedimentation of VLPs was confirmed by SDS-PAGE. For additional purification of VLPs, 300 µL of concentrated particles were transferred on 2.4 mL of 1.36 g/cm^3^ density CsCl solution in lysis buffer and separated at 75,000×g for 24 h at 4°C on the same ultracentrifuge with TST 60.4 rotor (Kontron). Fractions of 300 µL of the CsCl gradient were collected and analysed on SDS-PAGE. Fractions with the highest VLPs content were pooled and excess of salt was removed using Zeba spin 7K MWCO columns (Thermo Scientific), pre-equilibrated with lysis buffer following the manufacturer’s recommendations.

### 2.4 Purification of recombinant Gag and Gag-mCherry proteins from yeast

Synthesis of L-BC Gag and Gag-mCherry fusion protein was performed in *S. cerevisiae* BY4741 [LA-LBC-] cells using the expression vectors pFX7-LBC-gag and pFX7-LBC-gag + mCherry, respectively. Selection of yeast transformants was carried out on the YPD (2% peptone, 1% yeast extract, 2% glucose) agar plates (2% agar) supplemented with 0.024% formaldehyde. Transformed colonies were grown at 30°C in 250 mL YPD medium supplemented with 0.012% formaldehyde for 18–24 h. Recombinant protein synthesis was induced by changing the medium to 250 mL YPG induction medium (2% peptone, 1% yeast extract, 2% galactose) supplemented with 0.012% formaldehyde. After 16–18 h of induction, yeast cells were harvested by centrifugation at 1,000×g for 10 min at 4°C and frozen at −20°C, if purification was carried out later. Cells were resuspended in two volumes of lysis buffer (w/v) with 1 mM PMSF and disrupted by vortexing with double amount of 212–300 µm diameter glass beads (Roth) (w/w), ten times for 30 s with 30 s rests of the sample on ice. Cell debris and glass beads were removed by centrifugation at 1,000×g for 10 min at 4°C and soluble protein fraction was obtained by centrifugation at 15,000×g for 15 min at 4°C. The supernatant was diluted by lysis buffer to 15 mL and purified using the same ultracentrifugation methods as with bacteria-derived VLPs. Fractions with the highest VLP content were pooled, and excess of salt was removed using Zeba spin 7K MWCO columns (Thermo Scientific), pre-equilibrated with lysis buffer following manufacturer’s recommendations.

### 2.5 SDS-PAGE analysis

The protein samples were heated for 10 min at 95°C in reducing sample buffer (313 mM Tris-HCl pH 6.8, 10% SDS, 0.05% bromophenol blue, 50% glycerol, 0.06% β-mercaptoethanol) and separated in 8% SDS-PAGE gel using Mini PROTEAN^®^ Tetra Cell vertical mini gel electrophoresis system (Biorad) and PowePac™ HC High-current source (Biorad). Proteins were visualized by staining with 0.125% (w/v) Coomassie Brilliant Blue R-250 (Roth) in 50% isopropanol and 10% acetic acid solution.

### 2.6 Dynamic light scattering assay

DLS assay was employed to characterize the average size of the VLPs in solution. For the particle size stability and disassembly analysis, samples of purified VLPs were diluted to 0.5 mg/mL concentration with lysis buffer and centrifugated at 20,000×g for 5 min. For buffer exchange to storage buffers and solutions used in disassembly experiment, Zeba spin 7K MWCO columns (Thermo Scientific) were used following the manufacturer’s recommendations. The hydrodynamic diameter of VLPs was measured using Zetasizer µV (Malvern Instruments) instrument. Measurements were performed in triplicates (6 runs for each measurement) with a 30 s equilibration time between samples using a low-volume quartz batch cuvette (ZMV1002). The intensity of the scattered light was measured at room temperature with an angle of 90°.

### 2.7 Transmission electron microscopy imaging

For the visualization of L-BC VLPs, TEM imaging was performed. 3 μL of the sample was applied on carbon-coated copper grids (Agar Scientific, Stansted, United Kingdom), kept for absorbing for 1 min, dried with filter paper, and washed with 3 µL of sterile water for 10 s. The particles were stained with 3 µL of 2% aqueous uranyl acetate solution for 10 s, dried with filter paper, and additionally air-dried for 1 min. Electron microscopy of stained particles was performed using a Morgagni 268 (D) transmission electron microscope (FEI, Hillsboro, OR, United States).

### 2.8 Fluorescence thermal shift assay

FTSA experiments were performed using QIAGEN Rotor-Gene Q real-time PCR cycler (Qiagen). The optimal VLPs concentration was found to be 0.4 mg/mL. Fluorescence dye 1-anilino-8-naphtgalenesulfonate (ANS) solution in DMSO was added up to 100 µM final concentration. Samples of VLPs with dye were heated from 25°C to 99°C by increasing the temperature by 1°C per minute and measuring the reporter signal at each step. The fluorescence excitation and detection wavelengths were 365 and 460 nm, respectively. Particle TM values were obtained by fitting the melting curves to a two-state model using Thermott tool for FTSA data processing (Gedgaudas et al., 2022).

### 2.9 Packing of nisin into VLPs

The stock solution of antimicrobial peptide nisin Z (Handary S. A., Brussels, Belgium) was prepared at a molar concentration of 300 mM in water (1 mg/mL), filter-sterilized, and stored at 4°C. Before the encapsulation experiment, nisin was diluted with sterile water to molar concentrations of 220, 22 and 2.2 mM (0.74, 0.074 and 0.0074 mg/mL, respectively). Yeast-derived L-BC VLPs were prepared at molar concentration of 220 nM (or 2 mg/mL) in citrate-phosphate buffer (pH 6.0) with 300 mM NaCl and 20 mM EDTA using Zeba spin 7K MWCO columns. For the packing of nisin into VLPs, passive-diffusion method was used: solution of VLPs (220 nM) was mixed by equal volumes with nisin stock solutions (220, 22, 2.2 mM) to obtain mixtures of VLP:nisin with molar ratios of 1:1,000, 1:100 and 1:10 (1 mg/mL for VLPs and 0.37, 0.037 and 0.0037 mg/mL for nisin, respectively) in citrate-phosphate buffer (pH 6.0) with 150 mM NaCl and 10 mM EDTA. Particles were incubated with nisin overnight at 4°C with gentle shaking. Non-encapsulated nisin was filtered and washed from nisin-loaded VLPs using ultrafiltration columns (Pierce™ Protein Concentrators, 100 kDa cut-off, Thermo Scientific) following the manufacturer’s recommendations.

### 2.10 Agar-diffusion assay

The antimicrobial activity of VLP:nisin particles was determined by the agar diffusion assay using *B. subtilis* ATCC 6633, *Staphylococcus aureus* ATCC 29213, and *Streptococcus pyogenes* ATCC 19615 strains as gram-positive bacterial indicators. 20 μL of the VLP:nisin particles, VLPs without nisin (negative control), nisin in citrate-phosphate buffer (positive control), and unbound filtrated nisin samples were spotted on the LB agar plates seeded with overnight grown *Bacillus subtilis* and *S. aureus* cultures, as well as on the Tryptic soy agar medium (1.7% peptone from casein, 0.3% peptone from soymeal, 0.25% glucose, 0.5% NaCl, 0.25% K_2_HPO_4_, 2% agar, pH 7.3) inoculated with *S. pyogenes* cells (2 × 10^7^ cells/plate). The growth inhibition zones were measured in quintuplicate on plates after 24 h of incubation at 37°C and data was represented as the mean ± SD (mm).

### 2.11 Statistical analysis

Quantitative results are represented as mean ± standart deviation (SD) of three independent replicates. Statistical differences among groups were obtained by one-way ANOVA followed by Tukey’s multiple pairwise comparison test, with statistical significance marked as follows: p ≤ 0.05 (*), p ≤ 0.01 (**), p ≤ 0.001 (***), p ≤ 0.0001 (****). Statistical analysis was performed using GraphPad Prism software, version 8.0.1 (GraphPad Software, United States).

## 3 Results

### 3.1 Preparation and characterization of L-BC virus-like particles

The full-length capsid protein-coding GAG gene of the native L-BC virus (amino acids 1–697) was amplified from the viral genomic RNA of *S. cerevisiae* BY4741 strain by reverse transcription and subsequent PCR with gene-specific primers. The fragment was ligated into the pET28a (Shilling et al., 2020) bacterial and pFX7 (Sasnauskas et al., 1999) yeast inducible expression vectors, ligation products were used to transform *E. coli* DH101 cells, and the transformants were spreaded on agar plates with the appropriate antibiotic. Colonies containing pET28a-LBC-gag and pFX7-LBC-gag constructs were selected by plasmid restriction analysis, and the integrity and orientation of the GAG gene were confirmed by DNA sequencing.

**FIGURE 1:**
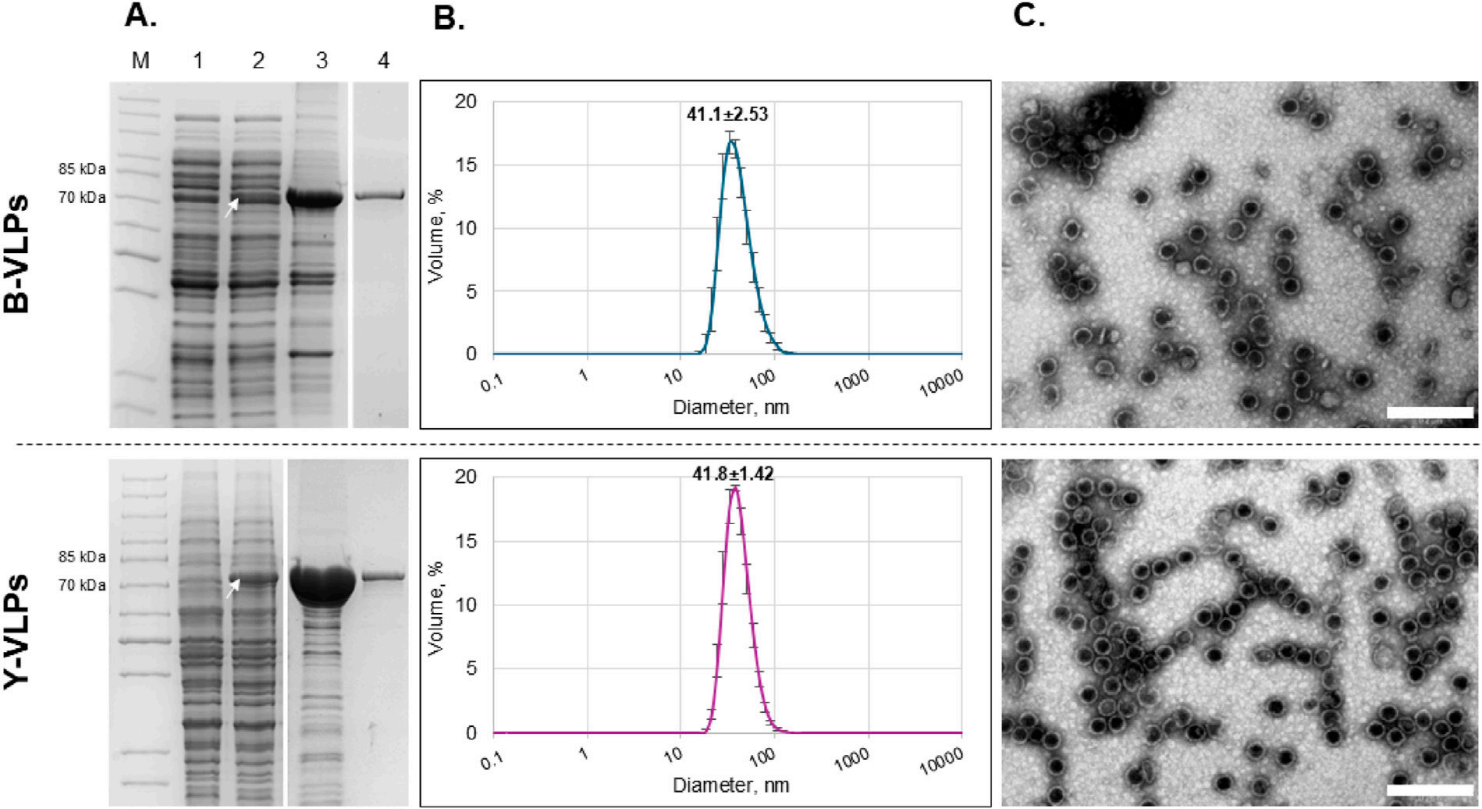
Characterization of L-BC Gag protein/VLPs purified from *Escherichia coli* BL21-AI (B-VLPs, upper panel) and *S. cerevisiae* BY4741 [LA-LBC-] (Y-VLPs, lower panel) strains. **(A)** SDS-PAGE analysis of protein samples. Lane M–PageRuler™ Unstained Protein Ladder. Lane 1 – soluble protein fraction obtained after induction and disruption of the cells transformed with pET28a (bacteria) and pFX7 (yeast) plasmids (negative control). Lane 2 – soluble protein fraction obtained after induction and disruption of the cells transformed with pET28a-L-BC-gag (bacteria) and pFX7-L-BC-gag (yeast) plasmids. Lane 3 – precipitate obtained after ultracentrifugation of soluble protein fraction through sucrose cushion. Lane 4 – a fraction with the highest amount of pure Gag protein obtained after purification in CsCl density gradient. The white arrow indicates the Gag protein (75–76 kDa). **(B)** Particle size distribution in the sample of purified Gag protein obtained by DLS. Data is displayed as percentage of the volume of three independent replicates with standard deviation. **(C)** Visualization of L-BC Gag VLPs using TEM. Scale-bare size: 200 nm.

For the synthesis of L-BC VLPs, pET28a-LBC-gag vector was transformed into *E. coli* BL21-AI strain and pFX7-LBC-gag vector was transformed into *S. cerevisiae* BY4741 [LA-LBC-] strain. After induction of the recombinant Gag protein expression, bacterial and yeast cells were disrupted by sonication or vortexing with glass beads, respectively. To confirm the synthesis of the Gag protein in both expression systems, soluble protein fractions were analysed in SDS-PAGE. A protein band of approximately 75–78 kDa corresponding to the calculated molecular mass of L-BC virus Gag protein (77.4 kDa) was present in both *E. coli* and *S. cerevisiae* samples (Figure 1A, lane 2). The recombinant Gag protein was concentrated from the total soluble protein fraction by ultracentrifugation through a sucrose cushion and resuspended in lysis buffer (Figure 1A, lane 3). Gag protein was additionally purified on the CsCl density gradient (Figure 1A, lane 4). Buffer exchange of the CsCl fractions with the most amount of Gag protein was performed on desalting columns before further processing. The total yield and purity of purified recombinant L-BC Gag protein were evaluated by densitometric analysis using ImageJ 1.53t tool (Schneider et al., 2012) The yield of Gag protein was 0.25 ± 0.03 mg/g (n = 4) of wet cells, or 0.55 ± 0.05 mg/L with 88%–98% purity for *E. coli* and 0.78 ± 0.05 mg/g (n = 3) of wet cells, or 6.69 ± 2.63 mg/L with 90%–92% purity for *S. cerevisiae*, as calculated for the highest concentration of product-bearing fraction.

To confirm the self-assembly of the recombinant L-BC Gag protein into VLPs and determine the average diameter of the particles in the samples, DLS and TEM analysis were performed. DLS measurements demonstrated that the average particle size in bacteria-derived samples (B-VLPs) was 41.1±2.53 nm, while in yeast-derived samples (Y-VLPs) – 41.8±1.42 nm (Figure 1B). TEM micrographs revealed the presence of empty symmetrical spherical structures with the average diameter of about 39–45 nm in both B-VLPs and Y-VLPs samples (Figure 1C). There was no observable difference in average particle size and morphology between the bacteria- and yeast-derived particle samples.

### 3.2 Size stability evaluation

**FIGURE 2:**
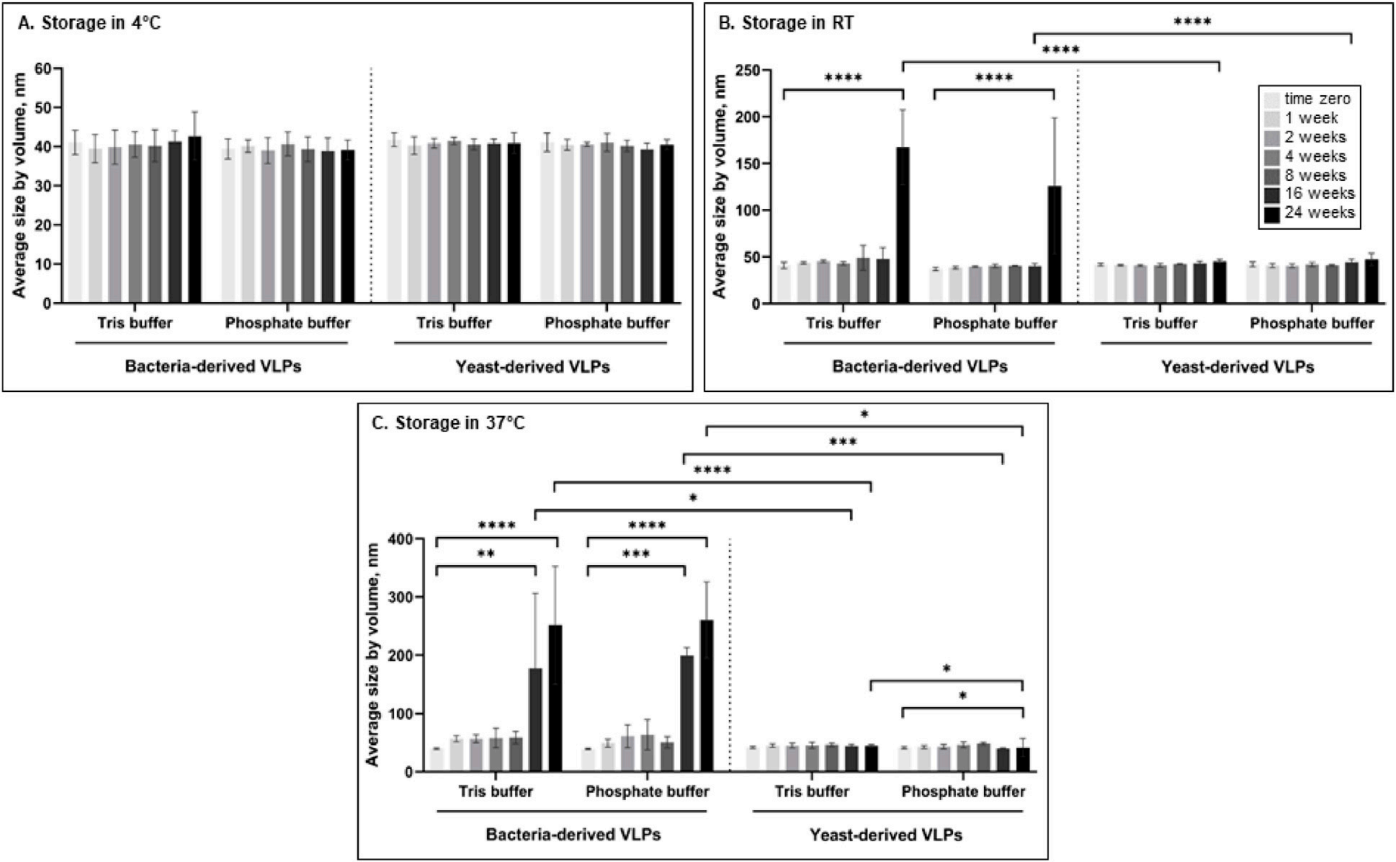
The average size of bacteria- and yeast-derived VLPs was measured by DLS incubating at 4°C **(A)**, RT **(B)** and 37°C **(C)** in Tris or phosphate buffer for up to 24 weeks. Error bars indicate the Standard deviation between three different independently purified particle batches. Statistical significance marked as follows: p ≤ 0.05 (*), p ≤ 0.01 (**), p ≤ 0.001 (***), p ≤ 0.0001 (****).

To evaluate and compare the stability of bacteria- and yeast-derived VLPs, the average size of the particles was measured by DLS during 24 weeks in different storage conditions. First, we evaluated the effect of temperature on the stability of L-BC VLPs in Tris- and phosphate-buffers (pH 8.0 with 150 mM NaCl and 10 mM EDTA) by measuring the average size of the particles at the time zero (T0) and after 1, 2, 4, 8, 16 and 24 weeks, incubating them at 4°C, 22°C (RT further on) and 37°C (Figure 2). When particles were stored at 4°C, no significant particle size change was observed throughout the 24 weeks in comparison with T0 in both B- and Y-VLP samples (p > 0.05) (Figure 2A). Yeast-derived particles that were stored at RT also observed no significant change in size (p > 0.05) throughout all experiments, however, there was an observable increase in the hydrodynamic diameter of bacteria-derived particles after 24 weeks of storage at RT compared to T0 (p ≤ 0.0001) in both Tris and phosphate buffers, indicating VLP aggregation (Figure 2B). After 16 weeks of the particle incubation at 37°C, there was a significant increase in bacteria-derived particle size in both buffers (p ≤ 0.01 for Tris and p ≤ 0.001 for phosphate buffer) and an even more significant increase in size after 24 weeks of incubation (p ≤ 0.0001 for both buffers). Yeast-derived particles observed better stability during the incubation at 37°C: particles that were incubated in Tris buffer had no significant change in size (p > 0.05) even after 24 weeks of incubation, and particles incubated in phosphate buffer demonstrated a slight decrease of the mean size at the end of the experiment (p ≤ 0.05) (Figure 2C). Storage of L-BC VLPs at different temperatures (4°C, RT, and 37°C) demonstrated, that yeast-derived particles are more stable than bacteria-derived particles, and in general, L-BC VLPs are more likely to aggregate during the incubation at higher temperatures. Also, most of the data indicate that there was no significant difference in the stability of VLPs in Tris or phosphate buffer (p > 0.05) except for Y-VLPs during incubation at 37°C for 24 weeks (p ≤ 0.05), so for further experiments phosphate buffer was chosen.

**FIGURE 3:**
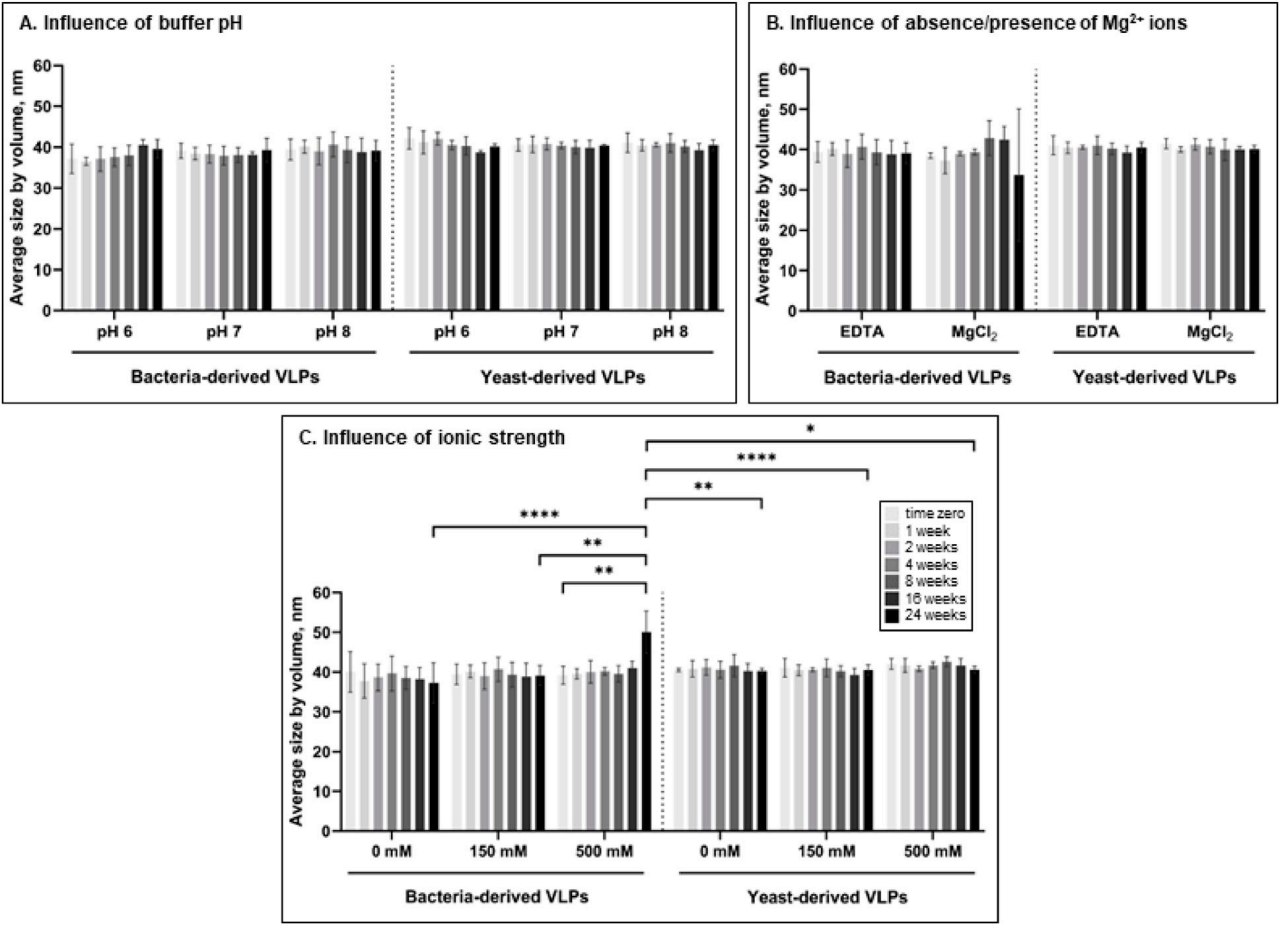
Impact of pH of phosphate buffer (150 mM NaCl, 10 mM EDTA) **(A)**, absence of Mg^2+^ ions (pH 8.0, 150 mM NaCl) **(B)**, and ionic strength (pH 8.0) **(C)** on the average size of bacteria- and yeast-derived VLPs by DLS for up to 24 weeks. Error bars indicate the standard deviation between three different independently purified particle batches. Statistical significance marked as follows: p ≤ 0.05 (*), p ≤ 0.01 (**), p ≤ 0.001 (***), p ≤ 0.0001 (****).

The influence of phosphate buffer pH, ionic strength (determined by NaCl), and presence of Mg^2+^ ions were evaluated during 24 weeks at 4°C by independent alteration of one of the conditions in the standard phosphate storage buffer (pH 8.0 with 150 mM NaCl and 10 mM EDTA) (Figure 3). Both bacteria- and yeast-derived VLPs were stable, with no significant change in size (p > 0.05) during the storage for 24 weeks at pH 6, 7, and 8 compared to T0 (Figure 3A). The presence of magnesium ions (10 mM) also had no significant effect on the average size of L-BC VLPs (p > 0.05) throughout the experimental period (Figure 3B). Yeast-derived VLPs had no significant change in size (p > 0.05) when stored in phosphate buffer with no added NaCl, physiological (150 mM) or high ionic strength (500 mM) additions. In the case of bacteria-derived particles, ionic strength of the buffer observed no or little impact on average size of the particles: significant change in size was observed only after 24 weeks of storage in buffer with high ionic strength compared to T0 (p ≤ 0.01) (Figure 3C).

### 3.3 Thermal stability of L-BC VLPs

**FIGURE 4:**
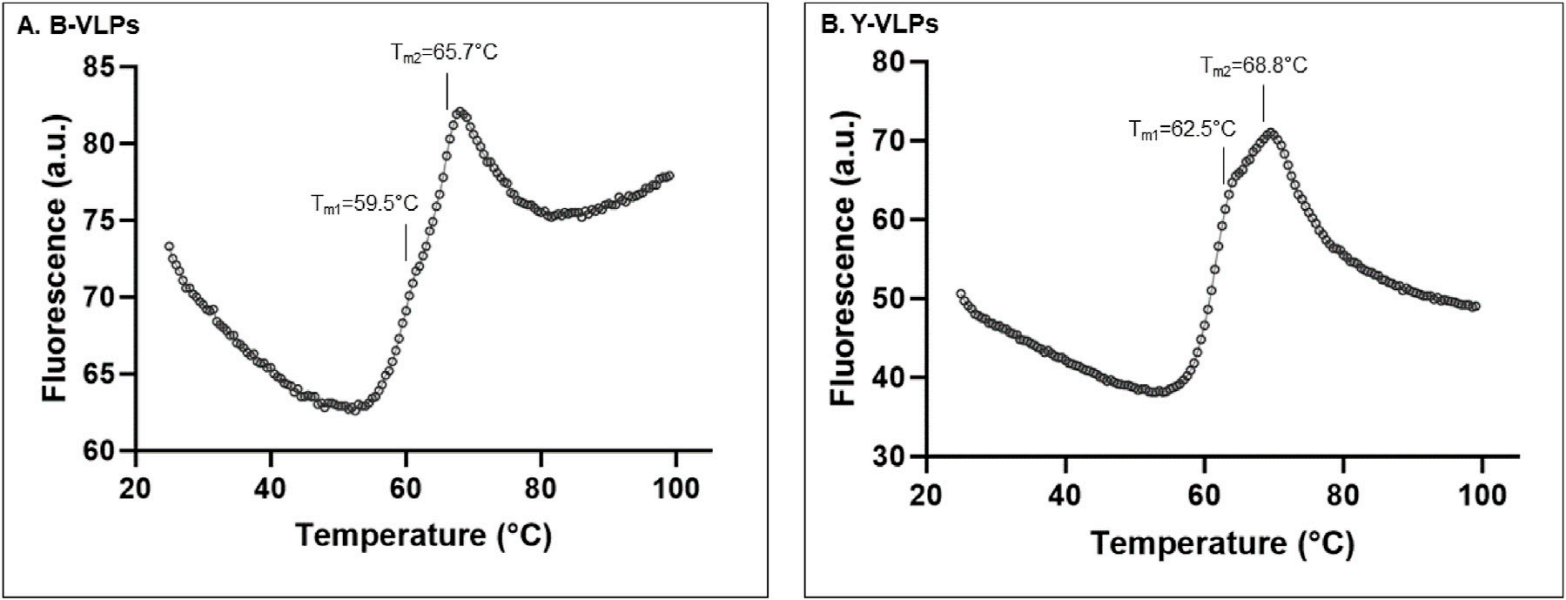
Thermal stability of B-VLPs **(A)** and Y-VLPs **(B)** in phosphate buffer (pH 8.0, 150 mM NaCl, and 10 mM EDTA) by thermal shift assay; a.u. – arbitrary units.

To better understand the impact of different temperatures and pH on the stability of L-BC VLPs, a fluorescent thermal shift assay (FTSA) was employed. ANS fluorescent probe that binds with high affinity to the hydrophobic surface of proteins accompanied by an increase in fluorescence was used to address the aggregation and denaturation of proteins (Ota et al., 2021). Both types of L-BC VLPs observed two thermal transitions in phosphate buffer (pH 8.0, 150 mM NaCl, and 10 mM EDTA), where the first peak with a lower fluorescence signal partially overlapped with the maximum of the second peak (Figure 4). Thermal shifting profiles showed that B-VLPs undergo the first thermal transition at 59.5°C (T_m1_) and second 65.7°C at (T_m2_) (Figure 4A), while Y-VLPs - at 62.5°C (T_m1_) and 68.8°C (T_m2_) (Figure 4B),. Thermal stability results correspond to our previous size stability data, where B-VLPs were more likely to aggregate at higher temperatures than yeast-derived particles.

**FIGURE 5:**
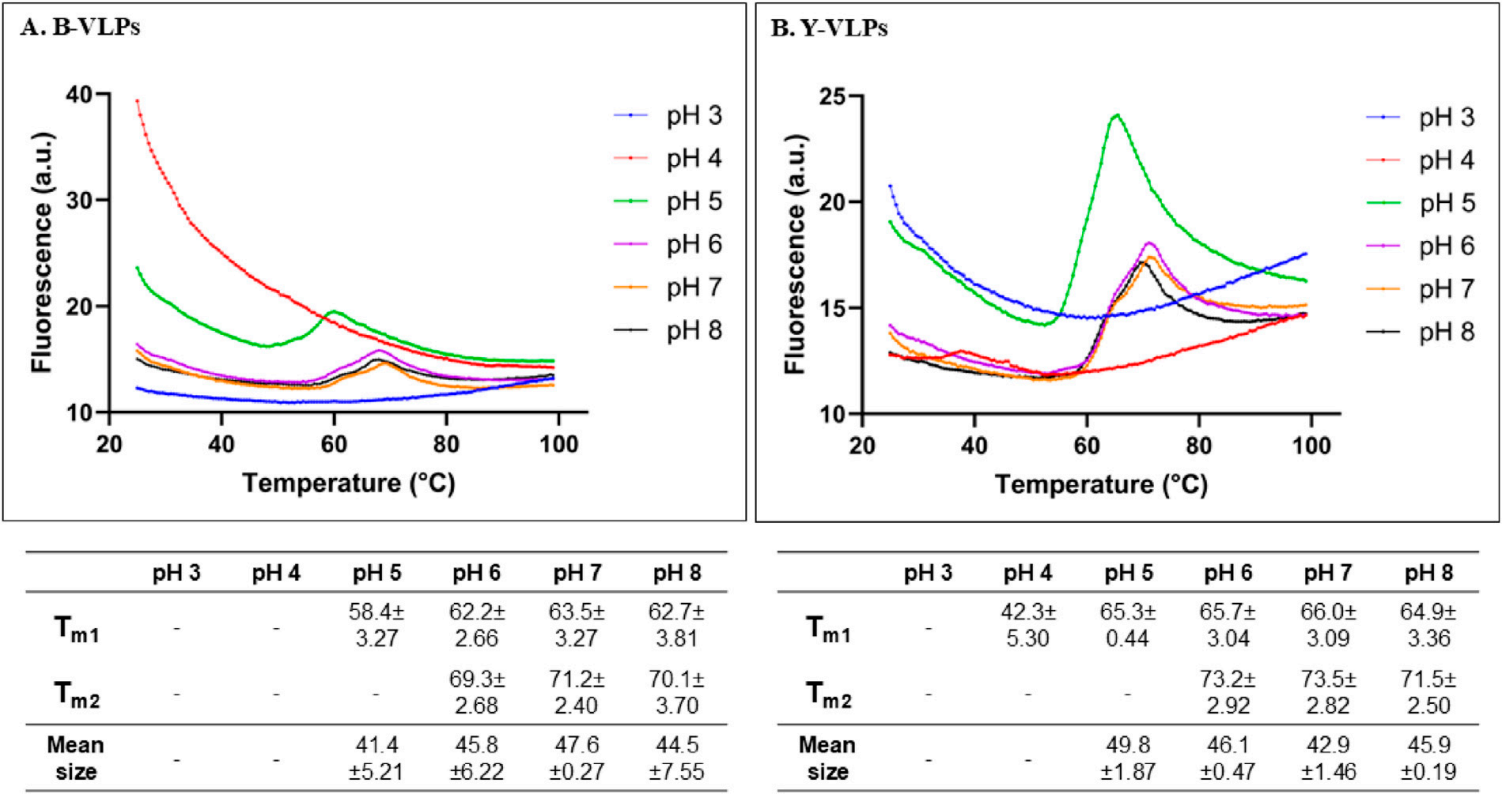
Thermal stability evaluation of L-BC VLPs in citrate-phosphate buffers (pH 3.0–8.0) by thermal shift assay and DLS: two shifting temperatures and average particle sizes of B-VLPs **(A)** and Y-VLPs **(B)**; a.u., arbitrary units. Shifting temperatures and size measurements are presented as mean with standard deviations between three replicates.

The pH-dependent thermal stability of B- and Y-VLPs was also investigated by thermal shift assay (Figure 5). To evaluate the thermal stability of L-BC VLPs in the wider range of pH (3.0–8.0), citrate-phosphate buffer system was chosen. At pH 3.0, both types of particles did not observe any thermal transition. B-VLPs also unfeatured thermal transition at pH 4.0, while Y-VLPs observe seemingly incidental thermal transition with low fluorescence intensity at 42.3°C. At pH 5.0, both types of particles demonstrated one shifting temperature, 58.4°C for B-VLPs and 65.3°C for Y-VLPs. Thermal stability of L-BC VLPs in citrate-phosphate buffer was quite similar in the pH range of 6.0–8.0 (64.9°C–65.7°C and 71.5°C–73.5°C for Y-VLPs and 62.2°C–63.5°C and 69.3°C–71.2°C for B-VLPs) (Figure 5); the particles featured two thermal transitions, as observed in phosphate buffer (Figure 4). The highest shifting temperatures were determined at pH 7.0: 63.5°C and 69.3°C for B-VLPs, 66.0°C and 73.5°C for Y-VLPs.

To better understand the observations from thermal shift experiments, DLS was employed to measure the average size of L-BC VLPs in solution at 25°C (Figure 5). DLS instrument did not detect any intact particles for both B- and Y-VLPs at pH 3.0 and 4.0. These data agree with the FTSA analysis and thus confirm the unfolded state of VLPs at a low pH environment. Surprisingly, at pH 5.0 already bacteria- and yeast-derived particles were intact with an average size of about 41.4–49.8 nm. At pH 6.0–8.0, the L-BC VLPs displayed a hydrodynamic diameter of 42.9–47.6 nm, a few nm larger than one measured in Tris and phosphate buffers. These observations are in a good agreement with the FTSA data confirming that at the starting point of 25°C of FTSA analysis, particles were intact and not dissociated.

### 3.4 Disassembly of yeast-derived L-BC VLPs

Our previous results revealed that yeast-derived L-BC VLPs possess a better tolerance to higher temperatures and ionic strength than bacteria-derived particles. To better understand the stability of L-BC VLPs, the average size of more stable yeast-derived particles was measured following the treatment with different agents for 16 h at 4°C to reveal the conditions that initiate particle disassembly (Table 2). As the first control, the size of particles in Tris buffer (pH 8.0, 150 mM NaCl, 10 mM EDTA) was measured (Table 2, No. 1) and the second control, particles in water without any additives, was also used (Table 2, No. 2). As observed earlier in our size stability experiments, Y-VLPs were insensitive to ionic strength changes in a range of 0–0.5 M NaCl, so higher concentrations of NaCl, particularly 1 and 2 M, with 5% of β-mercaptoethanol were tested for disassembly. After 16 h of particle incubation in Tris buffer with very high or no ionic strength and a high amount of reducing agent, no size changes were observed as compared with both controls (Table 2, No. 3–5). None of the previously tested conditions resulted in the dissociation of the yeast-derived particles, therefore urea was used as a denaturing agent for breaking down the capsid into individual proteins. Incubation of Y-VLPs in buffer with 1 and 2 M of urea led to no disassembly of the particles (Table 2, No. 6–7); however, upon incubation in buffer with higher amount of urea, 3 and 4 M, particles dissociated into ∼17.2 nm size structures (Table 2, No. 8–9). Interestingly, adding 5% β-mercaptoethanol into the buffer with 2 M of urea did not cause the breakdown of VLPs. The reducing and denaturing agents could have a synergistic effect and would stimulate the disassembly of VLPs. However, a small increase in hydrodynamic diameter of these particles was observed by DLS; it may be related to particle swelling or some other structural changes that may lead to further disassembly (Table 2, No. 10). Similar effect as with urea was observed when increasing pH from 11.0 to 12.0 using higher concentration of NaOH in water-particle mixture: at pH 11.0 and 11.5 average size of the particles was similar and comparable to that of controls, but when pH increased to 12.0 and 14.0, Y-VLPs disassembled into 13.8 nm size structures (Table 2, No. 11–14).

**TABLE 2:** Solutions with different agents were used to evaluate the disasembly of Y-VLPs and measured average sizes in the samples by DLS. Size results are presented as mean with standard deviations between three technical replicates.

No.	Solution			Size
1	100 mM Tris-HCl pH 8.0, 150 mM NaCl, 10 mM EDTA			42.5±1.96
2	Water			39.8±1.26
3	50 mM Tris-HCl pH 8.0, 5% βME		0 M NaCl	40.5±2.77
4			1 M NaCl	42.4±1.61
5			2 M NaCl	43.1±1.33
6	50 mM Tris-HCl pH 8.0		1 M urea	42.4±1.61
7			2 M urea	41.3±0.98
8			3 M urea	17.2±1.01
9			4 M urea	17.2±0.78
10	50 mM Tris-HCl pH 8.0, 5% βME		2 M urea	46.1±9.32
11	1 mM	NaOH	pH 11.0	40.3±0.73
12	7 mM		pH 11.5	35.6±1.58
13	10 mM		pH 12.0	13.8±0.60
14	30 mM		pH 14.0	13.8±0.54

**FIGURE 6:**
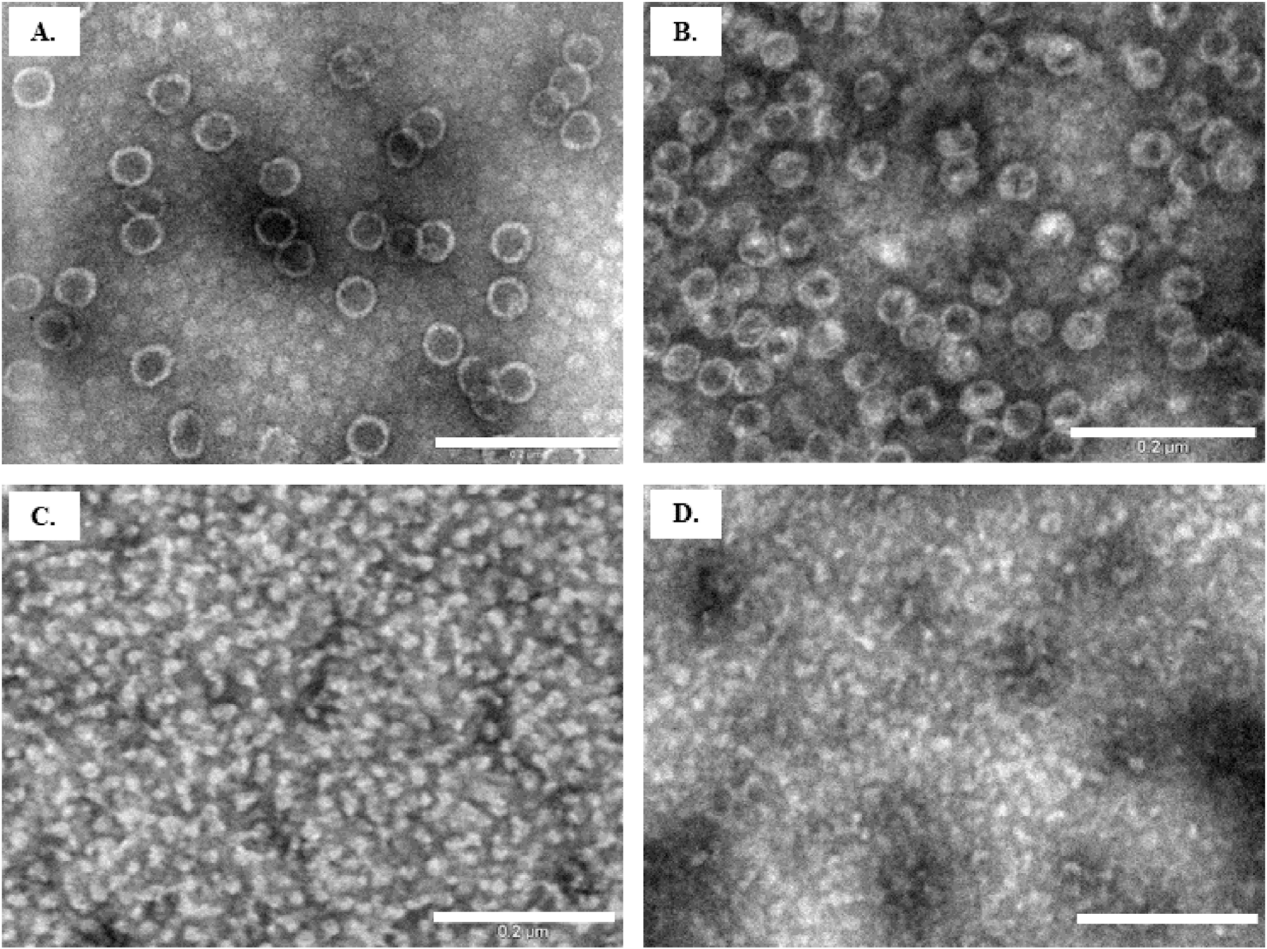
Evaluation of VLP disassembly by TEM. Particle samples after 16 h of incubation in **(A)** control buffer (solution No. 1); **(B)** buffer with 2 M NaCl (solution No. 5); **(C)** buffer with 3 M Urea (solution No. 8); **(D)** water with 10 mM NaOH (solution No.13). Scale bar size: 200 nm.

To confirm the disassembly of Y-VLPs, we also examined the treated samples by TEM (Figure 6). The average size of the particles in the control sample with standard lysis buffer (100 mM Tris-HCl pH 8.0, 150 mM NaCl, 10 mM EDTA) was about 45 nm. This diameter is comparable with the size revealed by DLS (Table 2, No.1 and Figure 6A). Particles incubated in a buffer with very high salt concentration (2 M NaCl) observed a similar size of about 42 nm. The high amount of salt in the sample may have altered the quality of the micrographs (Figure 6B). After 16 h of particle incubation in buffer with 3 M Urea, no intact VLPs could be identified, though unidentified structures in the size of 15–18 nm were seen, in good agreement with the DLS results (Figure 6C). Likewise, the incubation of particles in an alkaline environment (pH 12.0) resulted in the dissociation of Y-VLPs into similar size structures as shown in Figure 6D.

### 3.5 Encapsulation of mCherry protein into L-BC VLPs

**FIGURE 7:**
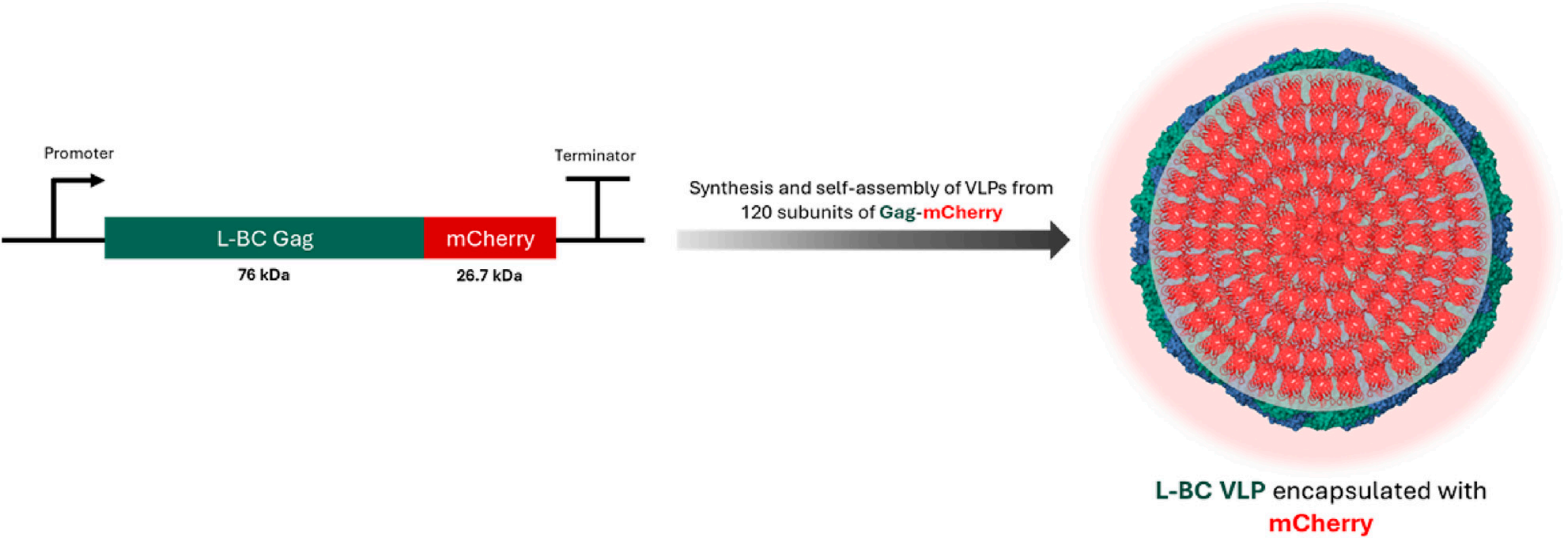
Schematic representation of encapsulation of mCherry protein into L-BC VLPs (PDB 7QWX) by gene fusion.

Following the observation of high endurance L-BC Gag VLPs at different conditions, we investigated the potential of L-BC VLPs to be employed as a nanocarrier. For this, we fused the red fluorescent protein mCherry to the C-terminus of the L-BC Gag protein, aiming to achieve the assembly of VLPs possessing 120 molecules of mCherry inside (Figure 7).

**FIGURE 8:**
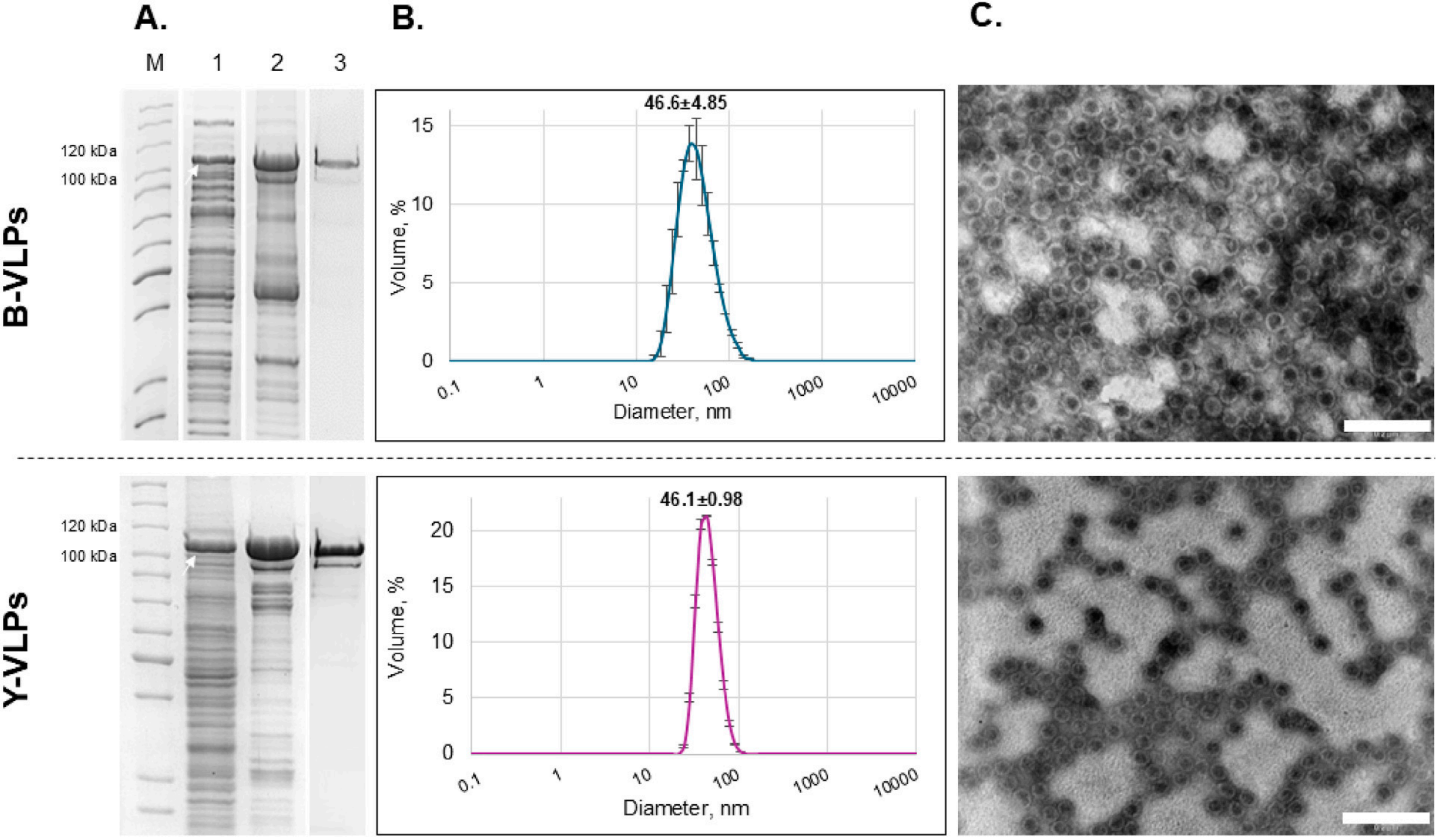
Characterization of L-BC Gag-mCherry fusion protein/VLPs purified from *Escherichia coli* BL21-AI (B-VLPs) and *S. cerevisiae* S2 (Y-VLPs) strains. **(A)** SDS-PAGE analysis of protein samples. Lane M–PageRuler™ Unstained Protein Ladder. Lane 1 – soluble protein fraction obtained after induction and disruption of the cells transformed with pET28a-L-BC-gag + mCherry (bacteria) and pFX7-L-BC-gag + mCherry (yeast) plasmids. Lane 2 – precipitate obtained after ultracentrifugation of soluble protein fraction through sucrose cushion. Lane 3 – a fraction with the highest amount of pure Gag-mCherry protein obtained after purification in CsCl density gradient. The white arrow indicates the Gag-mCherry fusion protein (∼103–105 kDa). **(B)** Particle size distribution in the sample of purified Gag-mCherry fusion protein obtained by DLS. Data is displayed as a percentage of the volume of three independent replicates with standard deviation. **(C)** Visualization of Gag-mCherry L-BC VLPs using TEM. Scale bar size: 200 nm.

For the synthesis of Gag-mCherry fusion protein, the same *E. coli* and *S. cerevisiae* strains that were previously employed for the generation of L-BC Gag VLPs, BL21-AI and BY4741 [LA-LBC-] respectively, were used. The successful synthesis of soluble Gag-mCherry fusion protein with the size of ∼103–105 kDa was confirmed after SDS-PAGE analysis of lysates obtained after induction of protein synthesis and lysis of both bacteria and yeast cells (Figure 8A, lane 1). The purification of Gag-mCherry fusion protein was carried out using the same approach as for Gag protein–sedimentation through sucrose cushion (Figure 8A, lane 2) and additional purification using CsCl density gradient (Figure 8A, lane 3). Successful self-assembly of VLPs was confirmed for both bacteria- and yeast-derived recombinant Gag-mCherry fusion protein using DLS and TEM assays. DLS analysis showed that the average particle size of Gag-mCherry VLPs, derived from bacteria and yeast, was 46.6 ± 4.85 and 46.1 ± 0.98 nm respectively (Figure 8B). Notably, these particles were about 5 nm bigger in diameter when compared to VLPs from wt Gag protein without mCherry. The size and morphology of Gag-mCherry particles in TEM micrographs were comparable to that of Gag VLPs, however, mCherry-encapsulated VLPs had a bit less symmetrical structure (Figure 8C). Despite that, Gag-mCherry fusion protein successfully self-assembled into VLPs in both bacteria and yeast cells, encapsulating 120 molecules of mCherry inside the particles without any noticeable effects on the particle structure.

### 3.6 Packing of nisin into yeast-derived L-BC VLPs by passive diffusion

**FIGURE 9:**
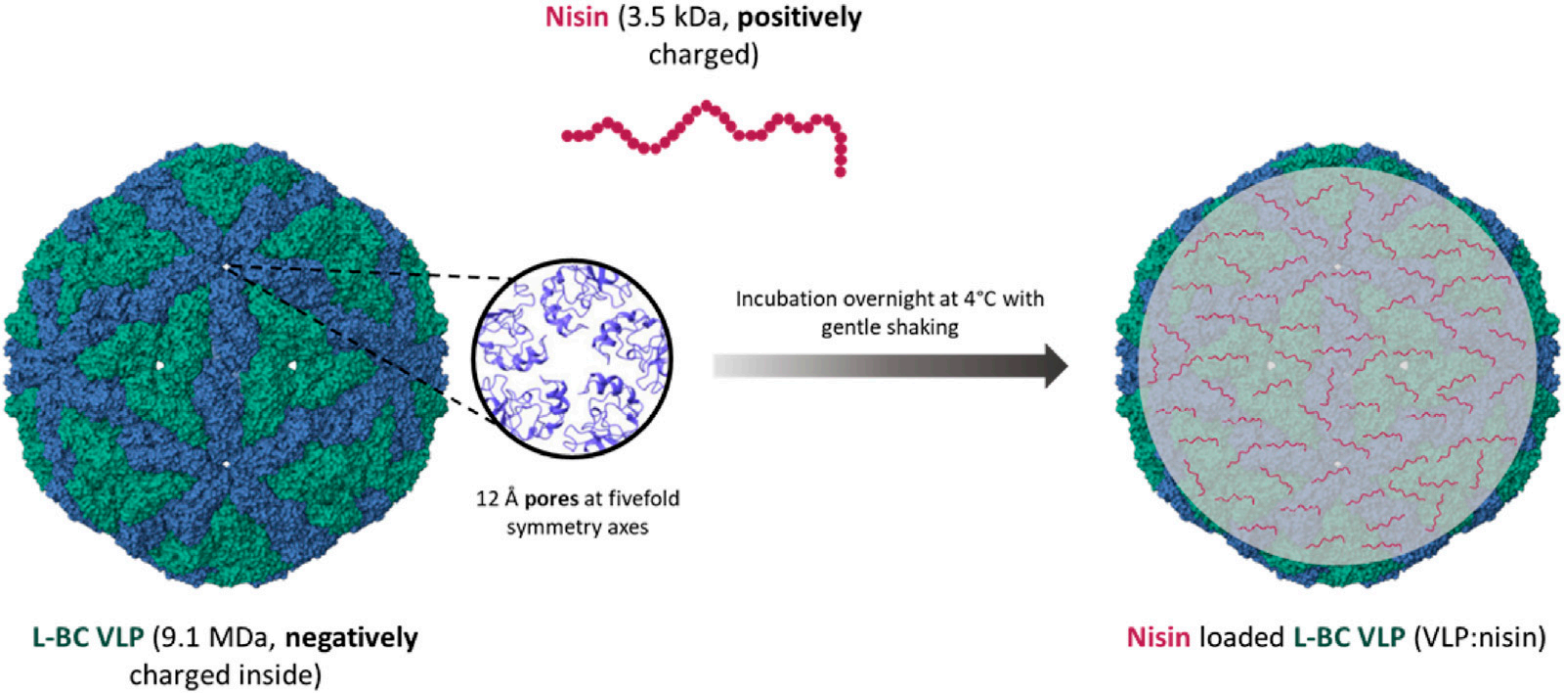
Schematics of nisin packing into the L-BC VLPs (PDB 7QWX) by passive diffusion through capsid pores.

To investigate the packing capability of L-BC VLPs, we also attempted to encapsulate positively charged antimicrobial peptide nisin Z into the negatively charged inner cavity of L-BC VLP. The passive diffusion approach was invoked, aiming at the pores, located in the fivefold symmetry axes (Figure 9).

**FIGURE 10:**
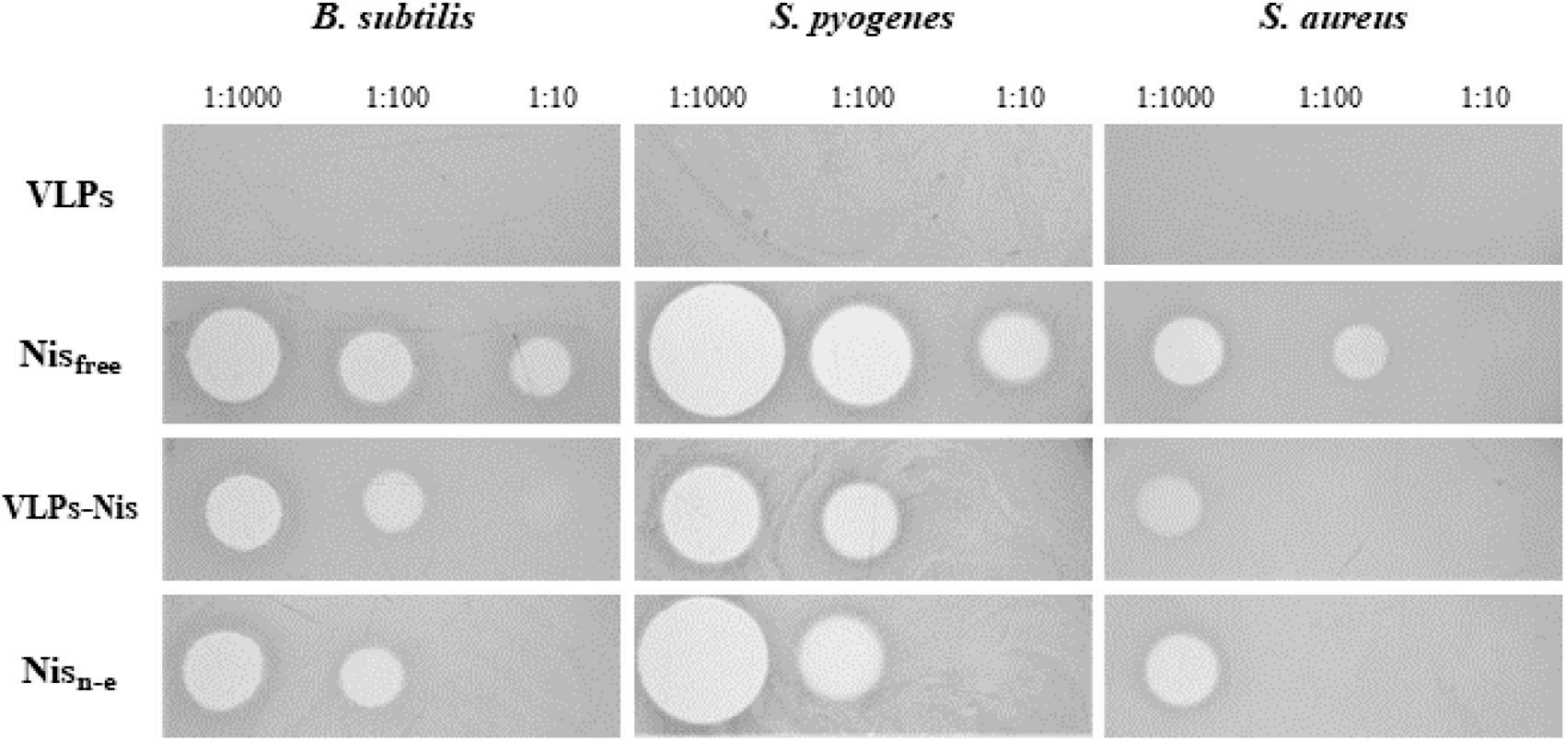
Bacteria viability to nisin-loaded VLPs. VLP - negative control, Nis_free_–free nisin (positive control), VLPs-Nis–nisin-loaded VLPs proceeded filtration and washing, Nis_n-e_–non-encapsulated nisin. The inhibitory activity was tested by measuring growth inhibition zones in an agar-diffusion assay. Numbers next to samples denote corresponding amount of nisin (in controls) or molar ratio of particles to nisin.

For the encapsulation of nisin, we chose yeast-derived L-BC VLPs, as observing better stability properties. Encapsulation was accomplished addressing three VLPs:nisin molar ratios: 1:1,000, 1:100 and 1:10. After diffusion-driven passive encapsulation, residual nisin was filtered and washed from the VLPs:nisin samples by re-concentrating on ultrafiltration columns three times. Using agar-diffusion assay, the antibacterial activities of VLPs:nisin, non-encapsulated nisin, free nisin (positive control), and VLPs (negative control) samples were tested against the Gram-positive *B. subtilis*, *S. pyogenes* and *S. aureus* bacterial strains on agar-diffusion assay (Table 3; Figure 10). As expected, L-BC Y-VLPs without nisin at a concentration of 1 mg/mL displayed no antibacterial activity on all three bacterial species tested. The filtered non-encapsulated nisin sample observed a slightly reduced activity profile compared to free nisin, pointing to the encapsulation of some nisin into the L-BC VLPs. Antibacterial activity of nisin-loaded L-BC VLPs with the highest (VLPs:nisin) molar ratio (1:1,000) was observed on all tested bacteria. The inhibitory effect of these samples was about 31%–42% lower than free nisin. Activity toward the *B. subtilis* and *S. pyogenes* strains was detected at 10-fold lower nisin concentration also (VLP:nisin molar ratio 1:100). Nisin-loaded VLPs samples prepared at 1:10 M ratio had no antimicrobial activity on any of the bacteria tested. We interpret so that the 1:10 M ratio is too low for efficient encapsulation of nisin into the VLPs. In general, *S. pyogenes* was the most sensitive to free or encapsulated nisin, compared to *B. subtilis* and *S. aureus* bacterial strains.

**TABLE 3:** Antimicrobial activity of nisin and nisin-loaded VLPs determined by agar-diffusion assay.

Sample		Growth inhibition zone, nm		
		*B. subtilis*	*S. pyogenes*	*S. aureus*
L-BC Y-VLPs (negative control)	0	0	0	0
Free nisin (positive control)	1,000 100 10	13.0 ± 0.4 9.9 ± 0.2 8.1 ± 0.2	20.4 ± 1.3 13.9 ± 1.2 9.7 ± 0.7	10.3 ± 0.4 8.2 ± 0.3 0
Nisin loaded L-BC VLPs	1:1,000 1:100 1:10	9.0 ± 0.8 8.1 ± 0.2 0	11.9 ± 0.5 10.2 ± 0.4 0	6.2 ± 1.1 0 0
Non-encapsulated nisin	1:1,000 1:100 1:10	12.2 ± 0.3 8.3 ± 0.4 0	19.9 ± 0.7 10.2 ± 0.4 0	9.6 ± 0.4 0 0

## 4 Discussion

Bacterial expression systems are routinely employed to produce of virus-like particles, such as originating from human papillomavirus type 52 (Mustopa et al., 2022), human parvovirus B19 (Sánchez-Rodríguez et al., 2012) or adeno-associated virus serotype 5 (Le et al., 2022), along with other VLPs. *S. cerevisiae* dsRNA virus L-BC was reported to lack prerequisite N-acetylation of capsid protein Gag for the assembly of virions (Wickner et al., 2013), therefore making virus-like particles suitable for production in *E. coli*. We invoked *E. coli* BL21-AI strain bearing the chromosome-located T7 RNA polymerase gene under the control of the arabinose-inducible araBAD promoter (Bhawsinghka et al., 2020), and took advantage of IPTG-inducible vector pET28a, constituting low background double-inducible expression system with high levels of capsid protein Gag expression. However, the yield of VLPs from *E. coli* was several times lower than that from *S. cerevisiae*; the prolongation of synthesis duration and increased concentration of inducers led to protein insolubility (data not shown). We also noticed growth inhibitory effects in *E. coli* expression system, pointing to possible toxicity of L-BC Gag protein to the bacterial cells, the precise mechanism of which remains to be elucidated.

To obtain viral genome-free VLPs, *S. cerevisiae* BY4741 strain was cured of both ScV-LA1 and ScV-LBC1 viruses originally present in this strain (Aitmanaitė et al., 2021). Elimination of viruses was confirmed by reverse transcription and PCR (data not shown). The yield of yeast system-derived Gag was found comparable to the yield of other virus-like particle proteins expressed in the same pFX7 expression vector – 0.85 ± 0.33 mg/g for tail tube protein of bacteriophage vB_EcoS_NBD2 (Špakova et al., 2019) and 0.42–1.05 mg/g for human polyomavirus HPyVs-derived VP1 proteins (Norkiene et al., 2015), albeit obtained using higher concentration of galactose (3%) than that of our experiments (2%). No adverse effects of L-BC Gag expression in the yeast system have been observed.

Formation of the bacteria- and yeast-derived L-BC VLPs was accessed by dynamic light scattering (DLS) and transmission electron microscopy (TEM) analysis. DLS is a simple and fast method evaluating the Brownian motion of the particles in solution and relating it with hydrodynamic diameter (Raval et al., 2019). This method is widely used for the evaluation of the size, aggregation and stability of VLPs in various conditions (Spice et al., 2020; Al-Ghobashy et al., 2017; Ausar et al., 2006; Hu et al., 2011; Lin et al., 2014). However, the DLS approach addresses the average size of the particles while omitting the examining of structural features, therefore for morphological analysis TEM was involved. Both approaches observed the average size of VLPs closely correlate with the previously reported diameter of the native L-BC virus - 38.5 nm (Grybchuk et al., 2022); of note, size of VLPs from both bacterial and yeast expression systems matches.

We took advantage of DLS to investigate the impact of different buffers, pH, ionic strength, and temperature on the size stability of L-BC VLPs. For our purification experiments we used Tris-HCl buffer (pH 8.0), while phosphate buffer provides better biocompatibility; therefore, both buffer systems were initially examined. Our data demonstrate that L-BC VLPs, both bacteria- and yeast-derived, feature the same size stability in Tris and phosphate buffers when incubating at 4°C, RT, and 37°C up to 24 weeks. A significant size increase of the yeast-derived particles was observed only after 24 weeks of incubation at 37°C, while bacteria-derived particles aggregated after 24 weeks during the storage at RT and after 16 weeks–at 37°C. For investigation of pH role in VLP stability, the citrate-phosphate buffer was involved. Surprisingly, the size of L-BC VLPs did not significantly change in the pH range of 6-8 in phosphate buffer with 10 mM of EDTA or Mg^2+^ ions. Yeast-derived particles were stable in phosphate buffer with 0–500 mM NaCl, though the size of bacteria-derived particles increased after 24 weeks of incubation in a buffer with a higher salt concentration (500 mM). A similar increase in size with temperature increase or elevation of salt concentration was observed during investigation of Canine parvovirus VLPs, explained by the increase of hydrophobic interactions in the inner layer of proteins that result in protein aggregation at high salt concentrations or temperatures (Xu et al., 2014). In general, L-BC VLPs exhibited profound stability in comparison to other VLPs. For example, experiments on the thermostability of human immunodeficiency virus (HIV) 1 envelope protein VLPs showed that after 1 week of storage at RT (24°C), particles started to form >300 nm aggregates in PBS (Aguado-Garcia et al., 2022). Norovirus GII-4 capsid VLPs were stable at RT (23°C) for up to 7 days in PBS pH 7.4 (Huhti et al., 2010). The identification of even trace amounts of aggregates is critical since their presence might result in an increased rate of aggregation during storage (Al-Ghobashy et al., 2017); we observe rather substantial stability of L-BC VLPs extending well beyond 1 year at 4°C (not shown) and up to 6 month at 37°C.

Thermal shift analysis of both bacteria- and yeast-originated L-BC VLPs revealed two transition temperatures with some ∼7.3°C difference, respectively. Of note is higher by ∼2.6°C transition temperatures of yeast-originated VLPs; given the natural host for capsid protein expression, this appears to reflect the property of the native condition. These findings point to the possible structural differences between bacteria- and yeast-derived particles, while the molecular peculiarities behind the increased stability, if any, remain to be determined. At pH 3.0 and 4.0, where both types of particles did not observe any thermal transition, the DLS analysis did not identify any intact particles. Here, low-temperature transition of 42.3°C observed for Y-VLPs at pH 4.0 appears as incidental due to negligible amplitude and rather irregular peak shape. Observation of undefined aggregates by DLS at pH 4.0 suggests that VLPs were already dissociated; therefore, observed thermal transition is merely related to the unfolding of CP. At pH 6.0–8.0, two transitions and intact particles were determined in both L-BC VLPs samples. Better thermal stability at pH 7.0 could be related to fact, that it mimics the condition in the cytosol, where fungal viruses persist due to the lack of an extracellular phase (Ghabrial et al., 2015).

Two thermal transitions of L-BC VLPs appear to relate to dissociation of VLPs (T_m1_) and subsequent unfolding of CP (T_m2_). In the thermal stability studies of Sweet potato feathery mottle virus (SPFMV) and Sweet potato mild mottle virus (SPMMV), two thermal shift transitions were also determined and linked with dissociation of the VLP into its constituent CP monomers (T_m1_) and the unfolding of the CP subunits (T_m2_) (Chase et al., 2023). For Bovine enterovirus type 2 (BEV2) and Equine rhinitis A virus (ERAV), similar to SPFMV and SPMMV viruses, the first transition is linked with the release of RNA, that could be related to the dissociation of virions, and the second transition reflecting the unfolding of CP (Walter et al., 2012). In thermal melting experiment on Norwalk virus VLPs, two shifting temperatures arise from the independent unfolding of two domains of particle-forming protein VP1 (Ausar et al., 2006).

For a better understanding of stability factors for L-BC VLPs and future application, the determination of conditions leading to the disassembly of these particles was performed. Some VLPs, for example, human papillomavirus type 11 (HPV-11) L1 VLPs, can be disassembled by incubating them with 5% reducing agent βME for 16 h at 4°C. These results demonstrated that disulfide bonds were essential for the stability of HPV-11 VLPs (McCarthy et al., 1998). Combination of ionic strength in Tris buffer (0, 1 or 2 M NaCl) with 5% of βME was insufficient for the disassembly of L-BC Y-VLPs. Urea is also often used for the disassembly of VLPs. The hepatitis B core protein VLPs can be disassembled in 2.5 M urea, whereas bacteriophage Qβ VLPs fully disassembled only in buffer with low ionic strength and 6 M urea (Le and Müller, 2021). In our experiments, 2 M urea was not sufficient for the disassembly of yeast-derived L-BC VLPs, and addition of 5% reducing agent (βME) did not led to disassembly of these particles–only the slightly increased their size. Complete disassembly of Y-VLPs was achieved only when urea concentration was higher than 3 M. Very high concentrations of urea can lead to the inability to reassemble VLPs from capsid proteins, so for reversable reconstitution it is advised to use an amount of urea just above the level needed for the stimulation of the dissociation of the particles. The alkaline environment is also one of the conditions for destabilization of virus capsids. Wild-type simian virus 40 (wtSV40) loses its infectivity and disassembles, when the pH of carbonate buffer is 10.7 or higher. It was determined that disassembly of this virus begins with the swelling of the particles and escaping of the genetic cargo through a formed hole in the capsid, followed by re-shrinking and complete disassembly of the virus capsid into subunits (Asor et al., 2020). pH 10 was found insufficient for the disassembly of yeast-derived L-BC VLPs, full decomposition was achieved when pH reached 12 and above.

The packing potential of L-BC VLPs was addressed first by conjugation of capsid protein Gag with red fluorescent protein mCherry. Since both N- and C-termini of protein are buried within a capsid, such approach poses a significant risk of structure overloading and subsequent rupture. The comparison of molecular masses of L-BC genetic material – 4.6 kb dsRNA, 4,600 × 700 Da–and a total number of mCherry – 120 × 26,000 Da–provides a reasonable background for genetic linking of Gag and mCherry. As follows from DLS and TEM observations, Gag-mCherry protein is capable for forming of stable particles, even though some 5 nm larger in diameter and somewhat affected overall symmetry. To the best of our knowledge, this is the first successful demonstration of loading by covalent linking of VLP with such proportion of cargo protein–up to a quarter of the total mass of particle. Notably, the resulting VLPs can be observed under daylight at concentrations below 0.1 mg/mL, while on fluorescent microscopy they visualize as an extremely bright particle.

Prompted by the prominent packing capacity, the diffusion-controlled loading was demonstrated by use of antibacterial peptide Nisin Z. L-BC capsids are recognized by the presence of pores, located at 5-fold symmetry centers, postulated to function as input gate for small molecules and output gate for RNA, synthesized within a virus particle. While RNA possesses a negative charge under the cellular pH value, nisin Z is charged positively, making it presumably unfavorable for packing within capsid. However, the inside surface of L-BC capsid was shown to be negatively charged (Grybchuk et al., 2022), allowing the successful loading of nisin Z. Loading of VLPs by nisin was found to be reversible, and determined the cargo-linked killing of sensitive bacteria.

## 5 Conclusion

In this work, we generated two expression systems for the synthesis of L-BC Gag VLPs in *E. coli* and *S. cerevisiae* cells. Our stability studies showed that these particles can tolerate various conditions, such as reasonably high or low salt concentration, elevated storage temperature (up to 37°C), acidic or alkaline environments, and reducing agents and stay intact in these conditions for a notably prolonged time. The successful encapsulation of an equimolar amount of mCherry protein, and encapsulation of antimicrobial peptide nisin Z by simple passive-diffusion method confirmed the high potential of particles in being developed into nanodelivery system. We are expanding this work by addressing the capabilities of VLPs to transfer cargo to mammalian cells, since the particles are stable enough to survive 37°C temperature for up to several months.

## Data Availability

The original contributions presented in the study are included in the article/Supplementary Material, further inquiries can be directed to the corresponding author.
