# Supplementary material for: Highly stable Saccharomyces cerevisiae L-BC capsids with versatile packing potential
Source: Front Bioeng Biotechnol. 2024 Sep 25;12:1456453. doi: 10.3389/fbioe.2024.1456453 (PMC11461329; doi:10.3389/fbioe.2024.1456453)
Supplement: Supplementary file 1 [file DataSheet1.PDF]

Supplementary Material

1 Supplementary Data

ScV-L-BC *GAG* gene

atgtcgtctctgttaaattcattactaccagaatattttaaacctaaaactaatttgaatatcaactcttctagggtccaatatggctttaatgctcgcattga
tatgcagtatgaagacgatagtgggactagaaaaggctcaagacccaatgcatttatgtctaacacagttgcttttataggaaactatgaaggtattat
tgttgatgacattccgatattggatggtcttagggccgacatttttgatactcatggtgacttagacatgggcctcgttgaggatgcattgtctaagagt
accatgattagaaggaatgtaccaacttatactgcttacgcgagtgaattactgtataagagaaaccttacatctctattttacaatatgctccgtttatac
tacattaaaaaatggggcagtattaagtatgaaaaagatgccatcttttatgataatggccacgcctgtcttttaaacaggcaattgtttccaaagtctc
gtgatgcttctttggaatcaagcctctctttacctgaggctgaaattgcaatgcttgatcctggcctggaatttccagaagaggatgtgcctgcaattta
tggcacggcagagtgtcatccagagcaacgtgtatcttagggcaagcttgctcagagttcgcgcctctggcccccttttcgattgcgcattattcacc
acaattgacgagaaaactatttgtcaatgcgcccgctgggattgagcctagctccgggcggtatactcacgaggatgtaaaagatgcgattacgat
ccttgtgtctgcaaaccaggcttatactgactttgaggcagcatacttgatgcttgctcaaacgttggtctcacctgtaccacgcactgccgaagcaa
gtgcatggttcatcaatgctggcatggtcaatatgccaactttgtcatgtgcaaatggttattatccagcactgaccaatgtcaatccttaccaccggct
agacacatggaaagatacgttaaatcattgggtggcttatcccgacatgctgttttaccattcagtggcaatgattgagagctgctatgttgaactcgg
gaatgtggctcgtgtgtcagacagtgatgcaataaacaaatacactttcactgagctatcagtgcaaggacggcctgttatgaatcgaggaattattg
tagatctgacacttgtggcaatgcgtactggtagggagatctcactaccttacccggtcagctgtggcctgacccgtacagacgcgttattgcaagg
tactgagattcacgttccagttgttgtcaaagatattgacatgccccagtattacaacgcgattgataaggatgttattgaggggcaggaaactgtgat
tagagtgaaacagctgccaccagctatgtatccaatttatacttacggcatcaacactactgaattctattctgaccattttgaagaccaggtacaagtt
gaaatggcaccaatcgataatggaaaagcagtttttaacgatgcaagaaagttttcgaaatttatgtccataatgcgcatgatggggaatgatgttact
gctactgatttagttacaggtagaaaagtgtcgaattgggccgacaactcatcagggcgtttcttgtacacggatgtgaagtatgaaggacaaactg
cctttttggttgatatggatactgtcaaggcgagagaccactgttgggtgtcaattgttgatcctaatggtacaatgaacttgtcatataagatgaccaat
tttagagcagcgatgttttctagaaacaagcccttgtatatgacagggggggtcagtcaggaccatagctactggcaattatcgagatgctgctgaaa
gattgcgtgcaatggatgaaacgctcagattaaaaccttttaagattactgagaagttggattttcgtgtagcagcttacgcgataccaagtttgtcgg
gcagcaatatgccatccttacaccatcaggaacaactacagatatcagaagtggacgcggaaccaatcaatcctataggagaggacgaacttcca
ccggatatagaa**tag**

ScV-L-BC *GAG* (blue) and *mCHERRY* (red) fusion gene

atgtcgtctctgttaaattcattactaccagaatattttaaacctaaaactaatttgaatatcaactcttctagggtccaatatggctttaatgctcgcattga
tatgcagtatgaagacgatagtgggactagaaaaggctcaagacccaatgcatttatgtctaacacagttgcttttataggaaactatgaaggtattat
tgttgatgacattccgatattggatggtcttagggccgacatttttgatactcatggtgacttagacatgggcctcgttgaggatgcattgtctaagagt
accatgattagaaggaatgtaccaacttatactgcttacgcgagtgaattactgtataagagaaaccttacatctctattttacaatatgctccgtttatac
tacattaaaaaatggggcagtattaagtatgaaaaagatgccatcttttatgataatggccacgcctgtcttttaaacaggcaattgtttccaaagtctc
gtgatgcttctttggaatcaagcctctctttacctgaggctgaaattgcaatgcttgatcctggcctggaatttccagaagaggatgtgcctgcaattta
tggcacggcagagtgtcatccagagcaacgtgtatcttagggcaagcttgctcagagttcgcgcctctggcccccttttcgattgcgcattattcacc
acaattgacgagaaaactatttgtcaatgcgcccgctgggattgagcctagctccgggcggtatactcacgaggatgtaaaagatgcgattacgat
ccttgtgtctgcaaaccaggcttatactgactttgaggcagcatacttgatgcttgctcaaacgttggtctcacctgtaccacgcactgccgaagcaa
gtgcatggttcatcaatgctggcatggtcaatatgccaactttgtcatgtgcaaatggttattatccagcactgaccaatgtcaatccttaccaccggct
agacacatggaaagatacgttaaatcattgggtggcttatcccgacatgctgttttaccattcagtggcaatgattgagagctgctatgttgaactcgg
gaatgtggctcgtgtgtcagacagtgatgcaataaacaaatacactttcactgagctatcagtgcaaggacggcctgttatgaatcgaggaattattg
tagatctgacacttgtggcaatgcgtactggtagggagatctcactaccttacccggtcagctgtggcctgacccgtacagacgcgttattgcaagg
tactgagattcacgttccagttgttgtcaaagatattgacatgccccagtattacaacgcgattgataaggatgttattgaggggcaggaaactgtgat

tagagtgaaacagctgccaccagctatgtatccaatttatacttacggcatcaacactactgaattctattctgaccattttgaagaccaggtacaagtt
gaaatggcaccaatcgataatggaaaagcagtttttaacgatgcaagaaagttttcgaaatttatgtccataatgcgcatgatggggaatgatgttact
gctactgatttagttacaggtagaaaagtgtcgaattgggccgacaactcatcagggcgtttcttgtacacggatgtgaagtatgaaggacaaactg
cctttttggttgatatggatactgtcaaggcgagagaccactgttgggtgtcaattgttgatcctaatggtacaatgaacttgtcatataagatgaccaat
tttagagcagcgatgttttctagaaacaagcccttgtatatgacagggggggtcagtcaggaccatagctactggcaattatcgagatgctgctgaaa
gattgcgtgcaatggatgaaacgctcagattaaaaccttttaagattactgagaagttggattttcgtgtagcagcttacgcgataccaagtttgtcgg
gcagcaatatgccatccttacaccatcaggaacaactacagatatcagaagtggacgcggaaccaatcaatcctataggagaggacgaacttcca
ccggatatagaagcggccgcgtccggagcaggtgctggtgctggtgctggagctagc**atg**gtgagcaagggcgaggaggataacatggccat
catcaaggagttcatgcgcttcaaggtgcacatggagggctccgtgaacggccacgagttcgagatcgagggcgagggcgagggccgcccct
acgagggcacccagaccgccaagctgaaggtgaccaagggtggcccctgcccttcgcctgggacatcctgtcccctcagttcatgtacggctc
caaggcctacgtgaagcaccccgccgacatccccgactacttgaagctgtccttccccgagggcttcaagtgggagcgcgtgatgaacttcgag
gacggcggcgtggtgaccgtgacccaggactcctccctgcaggacggcgagttcatctacaaggtgaagctgcgcggcaccaacttcccctcc
gacggccccgtaatgcagaagaagaccatgggctgggaggcctcctccgagcggatgtaccccgaggacggcgccctgaagggcgagatca
agcagaggctgaagctgaaggacggcggccactacgacgctgaggtcaagaccacctacaaggccaagaagcccgtgcagctgcccggcg
cctacaacgtcaacatcaagttggacatcacctcccacaacgaggactacaccatcgtggaacagtacgaacgcgccgagggccgccactcca
ccggcggcatggacgagctgtacaag**taa**
